# Effect of Cocoa Roasting on Chocolate Polyphenols Evolution

**DOI:** 10.3390/antiox12020469

**Published:** 2023-02-13

**Authors:** Alessandro La Mantia, Federica Ianni, Aurélie Schoubben, Marco Cespi, Klemen Lisjak, Davide Guarnaccia, Roccaldo Sardella, Paolo Blasi

**Affiliations:** 1 International School of Advanced Studies, University of Camerino, 62032 Camerino, Italy; 2 School of Pharmacy, University of Camerino, Via Madonna delle Carceri, 9, 62032 Camerino, Italy; 3 Department of Pharmaceutical Sciences, University of Perugia, Via del Liceo 1, 06123 Perugia, Italy; 4 Agricultural Institute of Slovenia, Department of Fruit Growing, Viticulture and Enology, Hacquetova ulica 17, SI-1000 Ljubljana, Slovenia; 5 Laerbium Pharma Srl, Via Togliatti n. 73/A, Corciano, 06073 Perugia, Italy; 6 Department of Pharmacy and Biotechnology, University of Bologna, Via San Donato 19/2, 40127 Bologna, Italy

**Keywords:** cocoa nibs, cocoa liquor, Folin–Ciocalteu assay, FRAP assay, chiral HPLC, catechin, epicatechin, mean degree of polymerization, Maillard reaction products

## Abstract

Cocoa and chocolate antioxidants might contribute to human health through, for instance, blood flow improvement or blood pressure and glycemia reduction, as well as cognitive function improvement. Unfortunately, polyphenol content is reduced during cocoa fermentation, drying, roasting and all the other phases involved in the chocolate production. Here, we investigated the evolution of the polyphenol content during all the different steps of chocolate production, with a special emphasis on roasting (3 different roasting cycles with 80, 100, and 130 °C as maximum temperature). Samples were followed throughout all processes by evaluating the total polyphenols content, the antioxidant power, the epicatechin content, and epicatechin mean degree of polymerization (phloroglucinol adducts method). Results showed a similar trend for total polyphenol content and antioxidant power with an unexpected bell-shaped curve: an increase followed by a decrease for the three different roasting temperatures. At the intermediate temperature (100 °C), the higher polyphenol content was found just after roasting. The epicatechin content had a trend similar to that of total polyphenol content but, interestingly, the mean degree of polymerization data had the opposite behavior with some deviation in the case of the highest temperature, probably due to epicatechin degradation. It seems likely that roasting can free epicatechin from oligomers, as a consequence of oligomers remodeling.

## 1. Introduction

Cocoa, obtained by processing the seeds *Theobroma cacao* L., and cocoa-derived products have been used for millennia and now we know that approximately 5500 years ago people leaving in Santa Ana-La Florida in the southeast part of Ecuador were already using cocoa [1,2]. Better known is the use of cocoa by Mayans and Aztecs that were used to consume it as unsweetened dark beverage [3].

Many beneficial or even curative effects have been reported for cocoa, chocolate, or other cocoa-derived products but only in recent decades, some of the positive effects claimed could be reliably demonstrated and scientifically validated. In fact, the improvement of analytical technologies and the development of more accurate analytical protocols have permitted the qualification and quantification of the numerous compounds responsible for these favorable effects. Many clinical studies have recognized that various of these effects are due to the presence of antioxidant compounds [4].

The most representative antioxidant compounds present in cocoa are catechins, anthocyanins, and proanthocyanins, among them, (-)-epicatechin is one of the most abundant flavanols and is also credited to be one of the key active molecules responsible for different biological activities.

Many biological effects with preventive or even curative potential on human pathologies have been reported [5]. For example, cocoa polyphenols, including (-)-epicatechin, may contribute to reducing blood pressure and glycemia during pregnancy [6], to improving cognitive function and reducing blood pressure in elderly with mild cognitive impairment [7], and have showed various disease modifying properties in patients affected by Alzheimer’s disease [8]. Some evidence on cocoa positive health effects against oxidative stress and chronic inflammation, risk factors for cancer and many chronic diseases, have been also reported [9].

Among the different biological effect reported, the most consolidated is the reduction of blood pressure after consumption of cocoa or cocoa-rich chocolate. In response to this evidence, in 2012, the European Food Safety Authority have issued a health claim related to cocoa flavanols and the maintenance of normal endothelium-dependent vasodilation by stating that the consumption of 200 mg/day of cocoa flavanols (provided by 2.5 g of high-flavanol cocoa powder or 10 g of high-flavanol dark chocolate), consumed in the context of a balanced diet, help to maintain endothelium-dependent vasodilation, which contributes to normal blood flow [10].

Cocoa generally contains a very high content of polyphenols per dry weight, much higher than other crops, foods, and beverages. For instance, tea and red wine, well known for their positive health effects ascribed to antioxidant compounds, have a lower content of polyphenols [11,12]. However, the large amount of polyphenols contained in fresh cocoa seeds, that fluctuates as a function of cocoa variety and pedoclimatic conditions, is significantly reduced as a consequence of the post harvesting processes (i.e., pod storage, fermentation [13], drying [14]) and of the manufacturing steps performed to obtain chocolate. Alkalization, roasting, and conching are among the steps more detrimental for the chemical integrity of cocoa polyphenols [15,16,17,18,19,20]. While alkalization is generally required in the production of cocoa powder and conching can also be avoided during the manufacturing of chocolate, roasting is the first step of chocolate production and is, virtually, always performed. Roasting and fermentation are the most important steps that allow the development of the taste and bouquet of the produced chocolate. On the other hand, these two steps are regarded as extremely detrimental for cocoa polyphenols and, generally, account for the loss of more than 50% of their initial polyphenol content [21].

Due to the important positive biological effects ascribed to cocoa polyphenols, that in some cases have been also clinically demonstrated, a deeper understanding of the fate of polyphenols during the whole chocolate manufacturing process is highly desirable. To this aim, cocoa beans were roasted using three different roasting temperature programs and the roasted cocoa was employed to produce Modica chocolate, *Cioccolato di Modica*. Modica chocolate was selected because of the mild production process (i.e., cold mixing of bitter cocoa paste with sugar) and does not contemplate the mixing at high temperature, the so-called conching [22]. All the manufacturing steps were monitored, the samples were analyzed at each step for their polyphenol content, epicatechin concentration, antioxidant capacity, and mean degree of polymerization of epicatechin oligomers.

## 2. Materials and Methods

### 2.1. Materials

(-)-Epicatechin standard, (±)-epicatechin, n-hexane, trifluoracetic acid (TFA), ethyl acetate (EtOAc), acetonitrile (ACN), formic acid (FA), anhydrous sodium sulphate (Na_2_SO_4_)_,_ pyridine, the derivatizing agent N,O-bis(trimethylsilyl)trifluoroacetamide (BSTFA), gallic acid, Trolox [(6-hydroxy-2,5,7,8-tetramethylchroman-2-carboxylic acid)], the Folin–Ciocalteu reagent, anhydrous sodium carbonate (Na_2_CO_3_)_,_ sodium acetate (CH_3_COONa), acetic acid (AcOH), ferric chloride (FeCl_3_), sodium sulphate, and hydrochloric acid (HCl) were purchased from Merck Life Science (Merck KGaA, Darmstadt, Germany). All the reagents were of analytical grade. Water for sample extraction was purified with a New Human Power I Scholar water purification system (Human Corporation, Seoul, Republic of Korea).

### 2.2. Cocoa Processing and Chocolate Preparation

Cocoa beans were processed to obtain the refined cocoa liquor by using a bean-to-bar equipment produced by the SELMI Group (Santa Vittoria d’Alba, Italy).

Fermented and dried cocoa beans (Cocoa beans) (6 Kg) were roasted in a Roaster 106 by using 3 different temperature programs (Table 1). Any roasting program was repeated 3 times obtaining 3 samples of 18 Kg of roasted cocoa beans (*Roasted cocoa beans*).

The roasted cocoa beans were then submitted to winnowing/cracking to obtain decorticated cocoa nibs (*Cocoa nibs*) that were grounded in particles of size between 200 and 250 μm (*Cocoa liquor*) (Stainless steel roller crusher, Grinder Mill, SELMI Group, Santa Vittoria d’Alba, Italy) and finally refined to a particle size between 20 and 25 μm (*Refined cocoa liquor*) (Micron ball refiner SELMI Group, Santa Vittoria d’Alba, Italy).

The refined cocoa liquor or cocoa paste were finally processed to obtain Modica chocolate. This particular type of chocolate was chosen because of the mild production process that does not contemplate the conching step that would have submitted the refined cocoa liquor to additional thermal stress. Modica chocolate was prepared by melting at a temperature ≤50 °C the refined cocoa liquor with sugar in the ratio 60/40 and mixing till obtaining a homogenous mass. The molten mass (100 g) was poured in a mold and left cooling down at a temperature ≤15 °C. Modica chocolate bars were produced by two chocolate makers of the *Consorzio di tutela del cioccolato di Modica*.

### 2.3. Sample Preparation and Polyphenols Extraction

Samples were grounded and sieved to obtain a powder with a particle size lower than 300 μm. Powdered samples (30 g) were dispersed in 150 mL of n-hexane and stirred for 3 h at room temperature. Afterwards, the suspension was filtered through a paper filter, washed with 50 mL of fresh n-hexane, and dried at room temperature. The defatted samples (0.5 g) were dispersed in 200 mL of extraction solution made up of MeOH/water/AcOH, in the ratio 79.2/20/0.8 (*v/v*), stirred for 3 h at room temperature, and filtered to obtain clear polyphenol solutions.

### 2.4. Determination of the Total Polyphenol Content with the Folin–Ciocalteu Assay

The total polyphenol content (TPC) of each extract was determined through the Folin–Ciocalteu assay as described previously with only few modifications [23,24]. The Folin–Ciocalteu reagent was diluted ten-fold with water and 0.1 mL of sample solution were added to 0.75 mL of reagent mixture and incubated for 10 min at room temperature in the dark. Afterwards, 0.75 mL of Na_2_CO_3_ (2%, *w/v*) were added, and the whole mixture incubated for 3 h at room temperature in the dark.

The absorbance was read at 765 nm with a UV-VIS spectrophotometer (Agilent 8453 spectrophotometer, Waldbronn, Germany). Gallic acid was used as a standard for the construction of the calibration curve, which was in the 1.275–12.75 μg/mL concentration range. All samples were analyzed in triplicate and the results obtained were expressed as milligrams of gallic acid equivalents per gram of defatted cocoa (mg GAE/g DC).

### 2.5. Determination of the Total Antioxidant Capacity by the FRAP Assay

Well aware that two main mechanisms can be at the basis of the antioxidant action of phenolic compounds, that is either through an electron transfer or a hydrogen transfer process (or a combination of both) [23,24], in the present study, the comparison among the total antioxidant capacity (TAC) of each extract was determined through the ferric reducing antioxidant power (FRAP) assay, as described previously with only slight modifications [23,24]. This basic procedure was considered suitable for the aim of the study. The FRAP reagent was prepared by mixing 2.5 mL of HCl 0.04 M with TPTZ (0.3%, *w/v*), 2.5 mL of FeCl_3_ (0.3%, *w/v*), and 25 mL of NaOAc (0.3 M, pH 3.6). A 1.5 mL aliquot of this mixture was added to 0.1 mL of water and 0.1 mL of polyphenol solution (see 2.4. sample preparation and polyphenols extraction) and incubated for 4 min at room temperature in the dark. The absorbance was read at 593 nm with a UV-VIS spectrophotometer (Agilent 8453 spectrophotometer, Waldbronn, Germany).

Trolox was used as a standard for the construction of the calibration curve, which was in the 0.59–5.88 μg/mL concentration range. All samples were analyzed in triplicate and the results obtained were expressed as milligrams of Trolox equivalent per gram of defatted cocoa (mg TE/g DC).

### 2.6. HPLC-UV Analysis for (-)-Epicatechin Quantitative Analysis: Experimental Conditions and Instrumentation

High-performance liquid chromatography analysis was carried out on a Shimadzu (Kyoto, Japan) LC-20A Prominence, equipped with a CBM-20A communication bus module, two LC-20AD dual piston pumps, a SPD-M20A photodiode array detector, and a Rheodyne 7725i injector (Rheodyne Inc., Cotati, CA, USA) with a 20 µL stainless steel loop. The obtained chromatogram was handled with the LC Solution Software from Shimadzu (Kyoto, Japan). The column temperature was fixed at 25 °C through a Grace (Sedriano, Italy) heather/chiller (Model 7956R) thermostat. The Grace Smart C18 column (100 Å, 5 μm, 4.6 mm i.d. × 250 mm, from Grace, Sedriano, Italy) was used for the HPLC analysis.

Before injection, each sample was filtered with a nylon 0.45 µm filter and analyzed in triplicate under gradient conditions. The mobile phase gradient program was obtained from eluent A [water/ACN/FA-95/4/1 (*v*/*v*/*v*)] and eluent B [water/ACN/FA-49.5/49.5/1 (*v*/*v*/*v*)] as follows: 0–10 min 10% B, 10–20 min up to 20% B, 20–30 min up to 80% B, 30–40 min up to 100% B. The eluent flow rate was fixed at 1.0 mL/min, while 275 nm was selected as detection wavelength.

The (-)-epicatechin content in the investigated extract samples was determined by relying upon a calibration curve built up in the 0.002–0.024 mg/mL concentration range (R^2^ = 0.998). To get the calibration curve, the concentration value of each epicatechin standard solution, analyzed in triplicate, was plotted against the corresponding area value.

**Figure 1:**
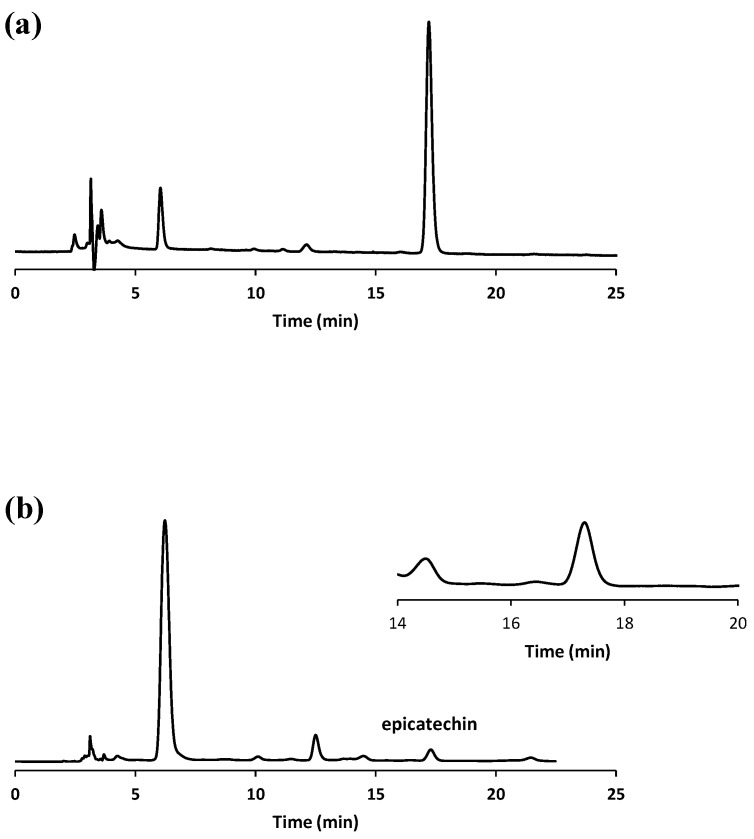
Representative HPLC chromatograms of (**a**) (-)-epicatechin standard solution and (**b**) an extracted sample (the magnified section of the chromatogram in the time-window containing epicatechin is highlighted).

In Figure 1, the HPLC chromatograms the of (-)-epicatechin standard solution and an exemplary extracted sample are shown.

**Figure 2:**
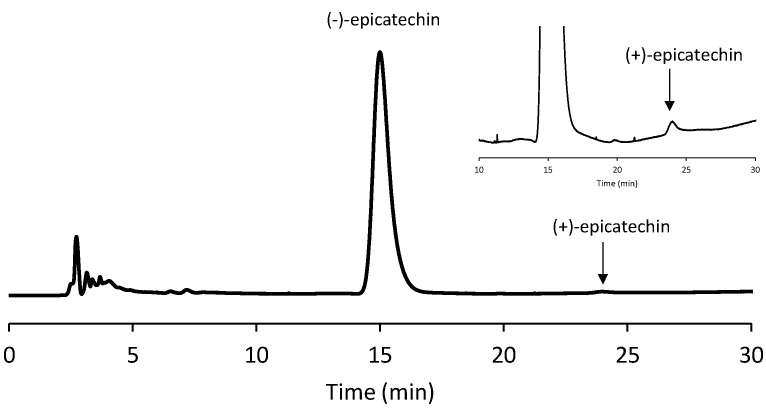
Representative enantioselective analysis of the peak corresponding to epicatechin in the achiral HPLC chromatogram. The magnified section of the chromatogram makes it evident the subtle presence of the (+)-epicatechin isomer.

The enantioseparation analysis was carried out slightly modifying a method found in the literature [25] with a Chiralcel OD-H column from Chiral Technologies (West Chester, PA, USA), containing cellulose tris(3,5-dimethylphenylcarbamate) adsorbed onto a 5 µm silica gel, as the chiral selector. Before the use, the selected column was conditioned with the employed mobile phase [n-hexane/EtOH/TFA–60/40/0.1 (*v*/*v*/*v*)] at a 1.0 mL/ min flow rate for at least 40 min. The LC enantioselective analyses were run at 1.0 mL/min flow rate with a 40 °C column temperature, setting the wavelength of detection at 275 nm. To confirm the isomeric identity of epicatechin, the corresponding peak in the achiral HPLC chromatogram was isolated from all the investigated samples, evaporated to dryness, and then analyzed with the above enantioselective LC conditions after solubilization in the selected eluent system. As a result, a predominance (>99.5%) of (-)-epicatechin was always detected, thereby justifying the exclusive consideration of this isomer throughout the manuscript. However, for a sake of simplicity, “epicatechin” will be used from now on in the manuscript. An exemplary chromatogram of a chiral analysis is shown in Figure 2.

### 2.7. GC-MS Analysis for (-)-Epicatechin Structure Confirmation: Experimental Conditions and Instrumentation

Gas chromatography-mass spectrometry (GC-MS) analysis was performed to ascertain the chemical identity of the HPLC peak attributed to epicatechin based on retention time correspondence with that of the reference standard. GC analysis of epicatechin standard, as well as of the corresponding peaks isolated in the HPLC runs of the extracts, was carried out after silylation reaction (see the following section for details). Analyses were performed on an Agilent 6850 Series Gas Chromatograph apparatus (Agilent Technologies, Santa Clara, CA, USA) fitted with a splitless injector for a low background HP-5MS fused silica capillary column (30 m × 0.25 mm i.d. × 0.25 μm film thickness) supplied by Agilent. A silanized injector liner split/splitless (2.0 mm i.d.) was used. Detection was carried out with a 5975B Mass single quadrupole spectrometer (Agilent Technologies). The GC–MS operation control and the data process were carried out by ChemStation software package (Agilent Technologies). The injector temperature was 250 °C. The temperature gradient program of the GC analysis was applied based on a previously developed and optimized method [26]. The oven temperature was held at 90 °C for 1 min, then increased to 220 °C at a heating rate of 6 °C/min, then to 290 °C at 10 °C/min and held for 1.23 min, and finally to 310 °C at a rate of 40 °C/min and the temperature was held for 7.5 min. The total run time was 37.67 min. The detector temperature was 280 °C. The carrier gas used was helium at a flow rate of 1.0 mL/min. The injected sample volume was 1.0 μL. The conditions for electron impact ionization (EI) were an ion energy of 70 eV and the mass range scanned was 140–600 *m*/*z*. The epicatechin-derivative fragmentation pattern was confirmed by comparison with data reported in literature [27].

### 2.8. Sample Derivatization Procedure for the GC-MS Analysis

**Figure 3:**
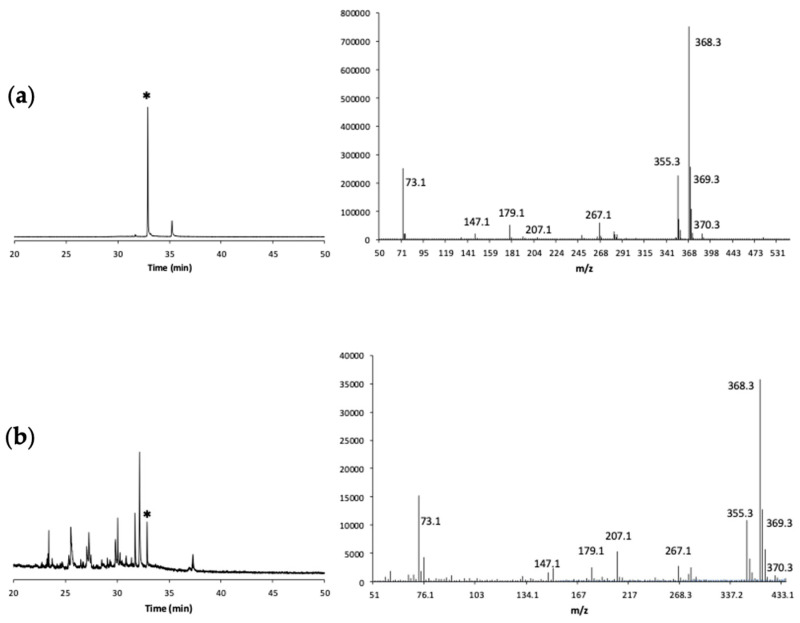
Representative GC chromatograms and MS spectra, in EI mode at 70 eV, of derivatives of (**a**) epicatechin standard and (**b**) epicatechin in a selected real sample. The epicatechin peak is marked with an asterisk.

A mixture of BSTFA and pyridine in ethyl acetate was used as the silylation reagent. One milliliter of each extract sample (in ethyl acetate or diethyl ether) was evaporated to dryness and transferred to a microvial for the GC-MS analysis [28]. Moisture is a major competitor of phenolic hydroxyl groups during derivatization with the mixture BSTFA-pyridine thus negatively affecting the derivatization efficiency. To avoid this problem, all reagents used for the silylation reaction were subjected to a dehydration pre-treatment with anhydrous sodium sulphate, which is effective in providing water-free conditions. The solid sample was dissolved in 0.2 mL of anhydrous ethyl acetate and the vial was stoppered. The derivatizing mixture BSTFA-pyridine-ethyl acetate (4/1/5, *v*/*v*/*v*) (0.5 mL) was added and the whole solution was mechanically shaken (through a vortex mixer) for 1 min at room temperature. Before injecting, samples were further diluted with 0.25 mL ethyl acetate (0.5 mL total volume). The results of a GC-MS analysis are reported in Figure 3. According to literature [26,28], the characteristic fragmentation patterns of the Trimethylsilyl (TMS) derivative of epicatechin are readily evident in the MS spectra displayed in Figure 3: *m*/*z* values of 73, 147, 179, 267, 355, 368, 369, 379.

### 2.9. Oligomers Extraction

Defatted samples (2.5 g) were added to 22.5 mL of acetone/H_2_O (80/20 *v/v*) solution and stirred for 4 h in an automatic shaker. After shaking, the mixture was centrifuged for 15 min at 5000 rpm. MeOH/H_2_O (60/40 *v/v*) (22.5 mL) was added to the defatted cocoa/chocolate powder left from the acetone extraction and shacked for 3 h. The mixture was centrifuged for 15 min at 5000 rpm and the methanol supernatant was added to the acetone supernatant. Acetone/MeOH/H_2_O extract was dried under reduced pressure. The dry residue was recovered with 25 mL of H_2_O, frozen, and freeze dried [29,30].

### 2.10. Mean Degree of Polymerization

Fractionation of cocoa proanthocyanidins and analyses of the reaction products were performed as described in Lisjak et al. [31]. Briefly, the oligomers extract (5 mg) was dissolved in 1 mL of MeOH, and 200 μL of this mixture was added to 200 μL of reaction solvent (10 g of phloroglucinol, 0.2 g of ascorbic acid and 0.167 mL of HCl 37 % (*v/v*) in 20 mL of MeOH) and left to react for 20 min at 50 °C. To quench the reaction, 1 mL of a CH_3_COONa water solution (0.04 M) was added to the mixture. The quenched reaction mixture was analyzed with HPLC-DAD-MS.

A HPLC Infinity 1290 coupled with 6460 Agilent Triple Quadrupole was used for the analyses. The column was XTerra^®^ RP 18 (4.6 × 100 mm, 3.5 μm particle size from Waters). The analysis was performed in gradient with two solvents: solvent A: aqueous solution of acetic acid (1% *v/v*) and solvent B: MeOH. The gradient features are reported in Table 2. The results were elaborated with the Mass Hunter B.04.01 software.

### 2.11. Correlation Analysis

Pearson’s correlation test was performed to determine the correlation between epicatechin content and TPC or epicatechin content and TAC. The correlation coefficients and associated probability values (two-tailed) were calculated and a minimum level of significance of *p* < 0.05 was used.

### 2.12. Principal Component Analysis

Principal component analysis (PCA) was performed on the normalized data excluding those of the initial cocoa beans (a value of 100% for all the parameters considered would have negatively affect the PCA results). The analysis was carried out using the correlation matrix to standardize the scales through the software Minitab 18 (Minitab Inc., State College, PA, USA).

## 3. Results and Discussion

Samples of unroasted cocoa, roasted cocoa (3 different temperature programs), intermediate products, as well as chocolate were collected at each step of processing and analyzed for their TPC, TAC, epicatechin content, and mDP. In particular, seven samples [named: *Cocoa beans, Roasted cocoa beans, Cocoa nibs, Cocoa liquor, Refined cocoa liquor, and chocolate (Modica)*] for each roasting temperature program were analyzed.

**Figure 4:**
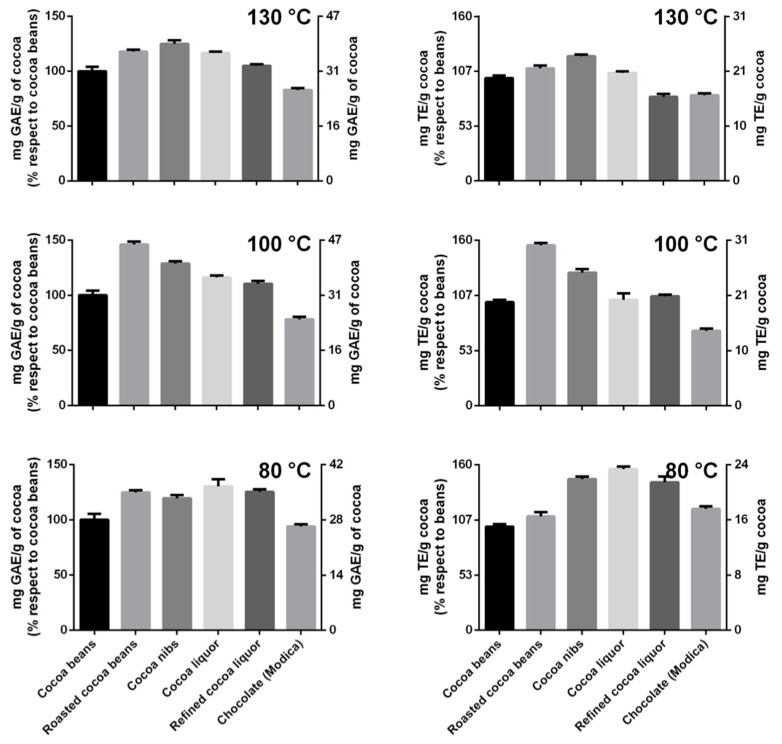
Data of TPC determined by Folin–Ciocalteu assay (**left** plots) and TAC determined by FRAP assay (**right** plots).

Interestingly, the polyphenol content, estimated by the Folin–Ciocalteu assay, when compared to that of unroasted cocoa beans, increased by different amounts in all the processing steps as a function of the roasting program, except for the last one. The intermediate temperature program was able to increase up to about 50 % of the TPC after roasting, while at lower or higher temperatures, the increases were less pronounced (Figure 4). The samples that reached 100 and 130 °C during roasting showed a TPC increase, followed by a reduction during the successive steps. The TPC of cocoa beans submitted to 80 °C remained almost stable after roasting (minor oscillations) with a sole reduction in the last step, originating a chocolate that had practically the same polyphenol content (~90%) of the original cocoa beans (Figure 4). Please note that in this specific case, the chocolate polyphenol content was referred to the grams of refined cocoa liquor employed in the formula and there is not a dilution effect due to the added sugar [32]. No other ingredients were added.

As expected, a similar trend was observed for the TAC, with the highest increase after roasting at the intermediate temperature program (maximum temperature reached, 100 °C) (Figure 4). The changes in antioxidant capacity of the cocoa submitted to 100 and 130 °C during roasting showed an astonishing similarity with the TPC data: an initial increase followed by a decrease. On the contrary, the TAC of the cocoa roasted at the lowest temperature (80 °C) had a slight increase during roasting although it continued to grow during the successive steps with a decline only at the two last steps. The produced chocolate had an antioxidant capacity slightly superior to that of the cocoa beans employed for its preparation (Figure 4).

The obtained results are somehow counterintuitive because it is well known that all the steps to produce chocolate, and especially those submitting the sample to thermal stress like roasting and conching, generally reduce the content of polyphenols and, consequently, the antioxidant capacity of the obtained product [33]. Indeed, Suazo and co-workers found that the higher is the temperature or the time of roasting, the higher is the loss of polyphenols [34]. However, the technique and the protocol employed for cocoa roasting may be optimized to reduce the detrimental effects on polyphenols, for instance superheated steam roasting has showed a lower impact on polyphenols than convectional roasting [35]. Air velocity and humidity during roasting have been seen also to have an impact on the extent of polyphenols decrease during the process [36].

Interestingly, the reduction of the TPC is not always accompanied by a reduction of the measured antioxidizing capacity that can even increase [32]. This evidence was explained by the fact that, while the TPC may decrease during roasting, some fractions of reducing substances may increase in concentration. Depending on the method used to quantify the free-radical scavenging activity, these fractions can contribute to a different extent to the TAC [37].

Even if a comparison with the literature data is not easy due to the different experimental setups, some authors reported a certain increase of TPC when roasting is performed at low temperature and short time [38,39]. In the present experimental set up, which was different from the majority of previous reports for the use of temperature ramps instead of fixed temperatures, it appeared readily evident that the intermediate temperature program provoked the highest increase of polyphenols/antioxidants but, overall, the best preservation of phenolic fraction and antioxidant capacity in the final product was guaranteed by the mildest temperature program.

A possible explanation on the rise of phenols/antioxidants is related to the contribution of new-born compounds, other than polyphenols, to the TPC due to the non-complete specificity of the polyphenol quantification assay. Here, TPC was obtained by using the Folin–Ciocalteu assay, a quick, cheap and versatile colorimetric method to quantify polyphenols in plant extracts and food matrices. The method is based on the transfer of electrons from phenolic compounds to phosphomolybdic/phosphotungstic acid complexes in alkaline environment. The reaction produces blue complexes that are quantifiable spectrophotometrically at a wavelength around 760 nm (765 nm in our protocol) [40].

Although this assay is a very useful analytical methodology for a rapid screening of large number of samples, the Folin–Ciocalteu reagent may react with other compounds giving rise to an underestimation or an overestimation of the phenolic fraction. In fact, it has been suggested to regard the Folin–Ciocalteu assay as a measure of the TAC rather than TPC [41]. In the specific case of cocoa and its derived products, due to the high abundance of phenolic compounds, the Folin–Ciocalteu assay may give a good approximation of the content of phenolic compounds [42].

The possible contribution of Maillard reaction products (MRPs) formed during roasting should be also considered. Indeed, during cocoa thermal treatment, a non-enzymatic reaction between reducing sugars and amino acids, peptides or proteins (i.e., the Maillard reaction), takes place, and a plethora of new compounds, referred as MRPs, is produced. High-molecular weight brown polymers, known as melanoidins, are a class of MRPs, produced during cocoa heat treatment that contribute to the color and flavor development. Melanoidins react with the Folin–Ciocalteu reagent and possess antioxidant activity, so it is reliable that, at least in part, the changes in the TPC and TAC values are due to the contribution of melanoidins and/or other MRPs developed during roasting and/or other production steps (e.g., grinding and refinement) that may produce local temperature increase in the sample [43,44,45].

It is also interesting to mention that cocoa melanoidins might contain polyphenols, such as epicatechins, either covalently bound in their backbone or adsorbed in them. It was shown that, upon partial hydrolysis, the released melanoidin-bound phenolic compounds maintain their scavenging properties [46].

**Figure 5:**
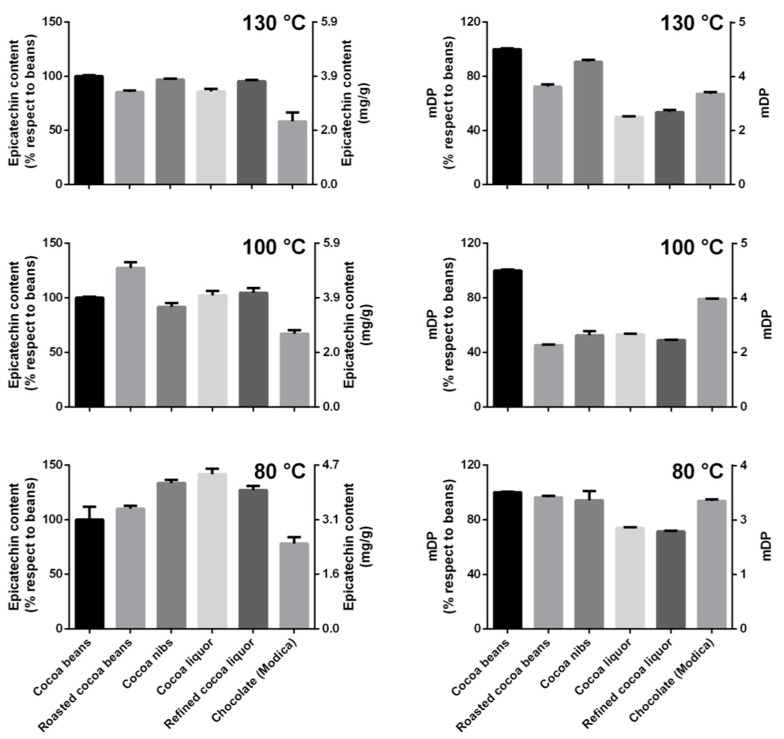
Epicatechin content determined by HPLC (**left** plots) and mDP data determined by using the phloroglucinol derivatization method (**right** plots).

Since epicatechin is one of the most abundant cocoa antioxidants, highly recognized for its beneficial effects in humans, the changes of epicatechin amount during the whole process of chocolate production were investigated (Figure 5). This information will also allow to envisage the contribution of epicatechin in the TPC and TAC.

Epicatechin content substantially increased during the chocolate production process, with a trend was very similar to that observed for the TAC, only when the cocoa beans were roasted through the mildest temperature program and the raw material did not exceed 80 °C. After roasting at intermediate/high temperatures, changes were present, but a clear trend could not be observed (Figure 5).

**Figure 6:**
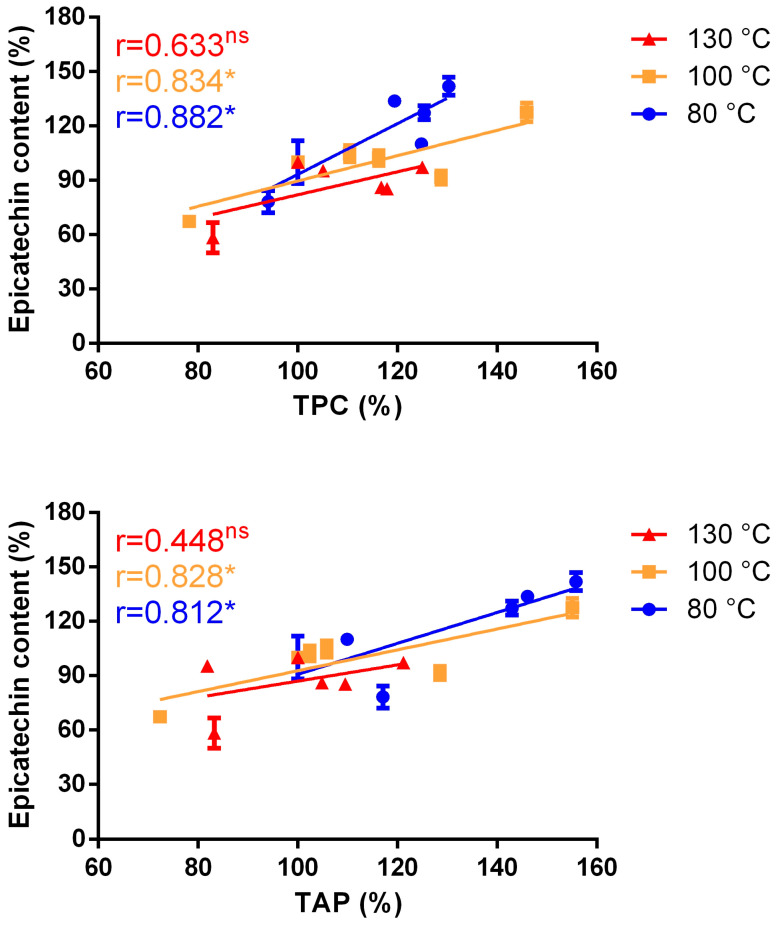
Correlation plots of TPC (%) and epicatechin (%) (**upper** plot) and of TAC (%) and epicatechin (%) (**lower** plot). Values have been normalized (100%) for the values of the relative cocoa bean sample. * significant (*p* < 0.05); ns, not significant (*p* > 0.05).

A Pearson correlation analysis between epicatechin content and TPC or epicatechin content and TAC revealed a significant correlation for low and intermediate temperature roasting programs, while no significant correlation was found for the highest temperature program (Figure 6), confirming the potential role of MRPs, in particular melanoidins.

These results seem to indicate that, at low and intermediate temperatures roasting programs, epicatechin contributes to the antioxidant activity of cocoa polyphenols. At higher roasting temperatures the absence of correlation suggests that other compounds, other than epicatechins, give a larger contribution to the matrix antioxidant capacity.

Overall, the obtained results tended to follow a similar trend: during roasting, and more in general during cocoa processing, the polyphenol content and the antioxidant capacity increased and then decreased to different extents, as a function of the roasting conditions. On the other hand, epicatechin showed the same behavior only when the mildest conditions were employed. While the first two experimental set ups (i.e., TPC and TAC) are indirect measurements and from these data it is not easy to give an interpretation at the molecular level, epicatechin concentration has been quantified directly by HPLC. So, the increase of epicatechin concentration during cocoa production can reliably be due to the release of epicatechin molecules from epicatechin oligomers as a consequence of roasting or all the other steps that submit the matrix to thermal and/or shear stresses.

**Figure 7:**
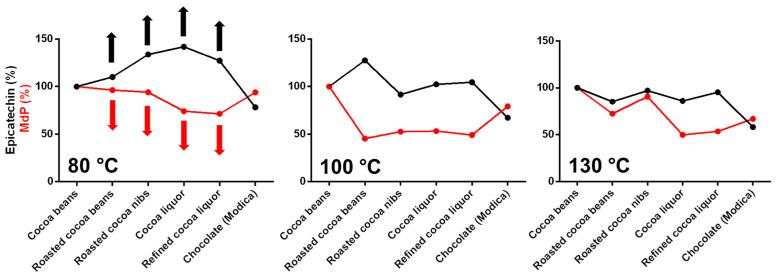
Comparison between the percentage of Epicatechin content (%) and mDP (%). Values have been normalized (100%) for the values of the relative cocoa bean sample.

To corroborate this hypothesis, oligomers were extracted and analyzed to calculate the mDP (Figure 5). A decrease of the mDP value during the different steps of chocolate production would indicate a reduction of the polymerization degree of the oligomers that might contemplate the release and/or the rearrangement of monomers, such as epicatechin. It is speculated that this mechanism was responsible for epicatechin variation. In particular, the cocoa samples submitted to the mildest temperature program had an increase in epicatechin content that corresponded to a decrease in mDP (Figure 5 and Figure 7). The same behavior was not observed for the samples treated at intermediate or high temperature.

Catechins oligomers, known as proanthocyanidins, has their own antioxidant capacity and their content in cocoa and chocolate was correlated to their antioxidant capacity [47]. Here, we report just the mDP, while their content was not evaluated; so, with the present experimental setup, it is not possible to draw conclusions on proanthocyanidin contribution to the antioxidant capacity of the analyzed samples. However, since the polyphenol extraction procedure was not optimized for proanthocyanidins, it is safe to state that their contribution was limited.

As previously stated, it is not common to find evidence of polyphenol increase during roasting or cocoa processing, but some reports support the finding that this increase can be due to the hydrolysis of oligomers [48,49]. In a recent report, Kothe and co-workers showed that dimmers and trimers can decrease and increase as a consequence of roasting and that the process is temperature dependent [48].

Although epimerization was considered the main mechanism of these variations, the rearrangements of oligomers cannot be excluded. Indeed, De Taeye and co-workers, in a very detailed investigation, clearly showed the possibility of oligomer rearrangement during roasting or the other steps for chocolate production. By studying the evolution of (−)-epicatechin and procyanidin B2 in aqueous and lipidic model media, the authors evidenced the formation of new oligomers, not present in the starting matrix. The same molecules (dimmers and trimers) were absent in unroasted cocoa beans but present after roasting, demonstrating the possibility of oligomers rearrangement, justifying the variations of mDP (decrease/increase) during the different steps of chocolate production and the possibility of monomer release during oligomer reorganization [50].

**Figure 8:**
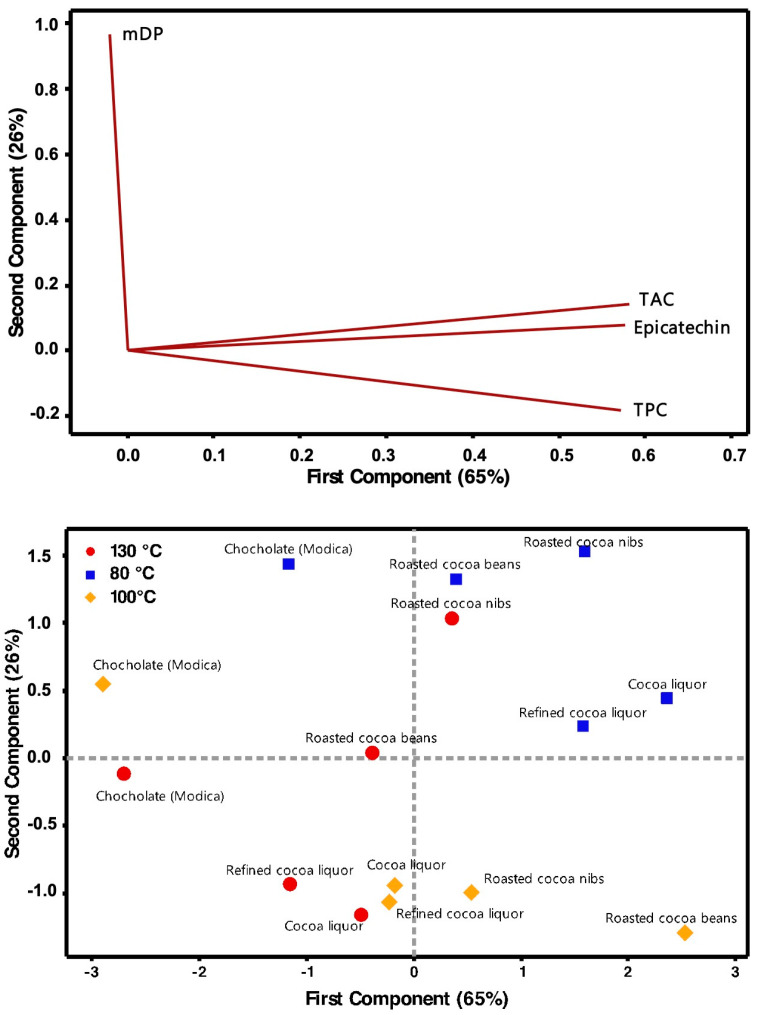
Loading plot (**upper**) and score plot (**lower**) representing the bidimensional space of PC1 and PC2 derived by PCA.

The PCA on the normalized data (excluding those of the fermented cocoa beans) can reduce the number of variables; in fact, the two principal components were able to explain more than 90% of the variation in the data. The variables contribute to the two eigenvectors in a very specific manner, in fact the first principal component depends mainly, and in a balanced manner (their coefficients in the eigenvector are all around 0.57), by TPC, TAC, and Epicatechin content, with the mDP responsible only for a marginal contribute (its coefficient in the eigenvector is −0.02). From the other side, the second principal component depends almost exclusively by the mDP (its coefficient in the eigenvector is around 0.87, while the second more relevant factor is TAC with a value of −0.19). These results allow an easy explanation of PCA eigenvectors, with the PC1 related to the polyphenols contents and antioxidant activity and the PC2 related the oligomerization degree. The effect of all the variables on the bi-dimensional space defined by the two principal components is reported in the loading plot (Figure 8 upper plot).

The final results of PCA are reported in the score plot (Figure 8 lower plot). The samples cluster according to their processing temperature. Samples treated at lowest roasting temperature are characterized by high values of PC1 and PC2, indicating an increase of polyphenols content and antioxidant activity, together with the oligomerization degree with respect to the initial samples. At intermediate temperature, there was still an average/high content of polyphenols and antioxidant activity, even if of less extent with respect to the sample treated at 80 °C, and a concomitant reduction of the oligomer length. Thus, the samples processed at 80 and 100 °C are well differentiated by the PC2. The samples processed at the highest temperature (130 °C) are instead clustered by the PC1 in a different plot area (lower left quadrant). In this case, all the samples showed intermediate or low values of PC1, indicating a reduction of the polyphenolic component.

Finally, it is interesting to note that the 3 chocolate samples did not cluster according to their processing temperature and seem to represent a separate group, characterized by low values of PC1 and high/intermediate values of PC2.

## 4. Conclusions

The results reported here have shown that roasting, and also the other steps needed for chocolate manufacturing, are responsible for the variation of the polyphenol content in the final product that is generally lower than that of the starting raw material. However, it is interesting to note that by employing real roasting programs, polyphenols and the antioxidant activity change by different extents as a function of the initial roasting program and the mildest conditions guarantee the best preservation of these healthy compounds. This investigation makes obvious the need for deeper and more accurate studies on the fate of the antioxidant compounds during chocolate manufacturing. In fact, most of the available reports concentrate their attention on a single manufacturing step or reproduce the single steps in the laboratory scale with conditions that may be substantially different from the real manufacturing conditions.

In conclusion, it is important to highlight that to have reliable indications on the preventive or curative effects described for cocoa/chocolate, a complete qualitative and quantitative characterization of the products is mandatory, especially when these are employed in clinical studies. At the same time, a deep characterization of the evolution of polyphenols (or other compounds of interest) during the whole production and manufacturing process will allow the optimization of the working parameters to obtain valuable and reproducible final products.

## Figures and Tables

**Table 1:** Parameters of the 3 different roasting programs.

Roasting Program	Temperature 1 (T_1_, °C)	Duration T_1_ (min)	Temperature 2 (T_2_, °C)	Duration T_2_ (min)	Maximum Temperature ^1^ (°C)
Low temperature	43	15	90	25	80
Intermediate temperature	95	10	130	15	100
High temperature	140	6	166	11	130

^1^ maximum temperature effectively reached by the sample during the roasting process.

**Table 2:** Gradient employed to separate fractionation products of proanthocyanidins extracted from cocoa.

Time (min)	Flow (mL/min)	Solvent B Percentage
0	0.7	5
25	0.7	5
45	0.7	20
60	0.7	32
62	0.7	100
67	0.7	100
68	0.7	5
72	0.7	5

## Data Availability

The data presented in this study are available on request from the corresponding author.

## References

[1] Lanaud C., Loor R., Zarrilo S., Valdez F. (2012). Origen de La Domesticacion Del Cacao y Su Uso Temprano En El Ecuador. Nuestro. Patrim..

[2] Zarrillo S., Gaikwad N., Lanaud C., Powis T., Viot C., Lesur I., Fouet O., Argout X., Guichoux E., Salin F. (2018). The Use and Domestication of Theobroma Cacao during the Mid-Holocene in the Upper Amazon. Nat. Ecol. Evol..

[3] Dillinger T.L., Barriga P., Escárcega S., Jimenez M., Salazar Lowe D., Grivetti L.E. (2000). Food of the Gods: Cure for Humanity? A Cultural History of the Medicinal and Ritual Use of Chocolate. J. Nutr..

[4] Ackar D., Valek Lendić K., Valek M., Šubarić D., Miličević B., Babić J., Nedić I. (2013). Cocoa Polyphenols: Can We Consider Cocoa and Chocolate as Potential Functional Food?. J. Chem..

[5] Magrone T., Russo M.A., Jirillo E. (2017). Cocoa and Dark Chocolate Polyphenols: From Biology to Clinical Applications. Front. Immunol..

[6] Di Renzo G.C., Brillo E., Romanelli M., Porcaro G., Capanna F., Kanninen T.T., Gerli S., Clerici G. (2012). Potential Effects of Chocolate on Human Pregnancy: A Randomized Controlled Trial. J. Matern. Fetal. Neonatal. Med..

[7] Desideri G., Kwik-Uribe C., Grassi D., Necozione S., Ghiadoni L., Mastroiacovo D., Raffaele A., Ferri L., Bocale R., Lechiara M.C. (2012). Benefits in Cognitive Function, Blood Pressure, and Insulin Resistance through Cocoa Flavanol Consumption in Elderly Subjects with Mild Cognitive Impairment: The Cocoa, Cognition, and Aging (CoCoA) Study. Hypertension.

[8] Wang J., Varghese M., Ono K., Yamada M., Levine S., Tzavaras N., Gong B., Hurst W.J., Blitzer R.D., Pasinetti G.M. (2014). Cocoa Extracts Reduce Oligomerization of Amyloid-β: Implications for Cognitive Improvement in Alzheimer’s Disease. J. Alzheimers. Dis..

[9] Maskarinec G. (2009). Cancer Protective Properties of Cocoa: A Review of the Epidemiologic Evidence. Nutr. Cancer.

[10] (2012). EFSA Panel on Dietetic Products, Nutrition and Allergies (NDA) Scientific Opinion on the Substantiation of a Health Claim Related to Cocoa Flavanols and Maintenance of Normal Endothelium-Dependent Vasodilation Pursuant to Article 13(5) of Regulation (EC) No 1924/2006. EFSA J..

[11] Arts I.C., Hollman P.C., Kromhout D. (1999). Chocolate as a Source of Tea Flavonoids. Lancet.

[12] Arts I.C., van de Putte B., Hollman P.C. (2000). Catechin Contents of Foods Commonly Consumed in The Netherlands. 1. Fruits, Vegetables, Staple Foods, and Processed Foods. J. Agric. Food Chem..

[13] Albertini B., Schoubben A., Guarnaccia D., Pinelli F., Della Vecchia M., Ricci M., Di Renzo G.C., Blasi P. (2015). Effect of Fermentation and Drying on Cocoa Polyphenols. J. Agric Food Chem..

[14] Guehi T.S., Zahouli I.B., Ban-Koffi L., Fae M.A., Nemlin J.G. (2010). Performance of Different Drying Methods and Their Effects on the Chemical Quality Attributes of Raw Cocoa Material. Int. J. Food Sci. Technol..

[15] Jalil A.M.M., Ismail A. (2008). Polyphenols in Cocoa and Cocoa Products: Is There a Link between Antioxidant Properties and Health?. Molecules.

[16] Hii C.L., Law C.L., Suzannah S., Misnawi, Cloke M. (2009). Polyphenols in Cocoa (*Theobroma Cacao* L.). Asian J. Food Agro.-Ind..

[17] Redovniković I.R., Delonga K., Mazor S., Dragović-Uzelac V., Carić M., Vorkapić-Furač J. (2009). Polyphenolic Content and Composition and Antioxidative Activity of Different Cocoa Liquors. Czech J. Food Sci..

[18] Maldonado-Mateus L.Y., Perez-Burillo S., Lerma-Aguilera A., Hinojosa-Nogueira D., Ruíz-Pérez S., Gosalbes M.J., Francino M.P., Rufián-Henares J.Á., Cueva S.P. (2021). de la Effect of Roasting Conditions on Cocoa Bioactivity and Gut Microbiota Modulation. Food Funct..

[19] Di Mattia C.D., Sacchetti G., Mastrocola D., Serafini M. (2017). From Cocoa to Chocolate: The Impact of Processing on In Vitro Antioxidant Activity and the Effects of Chocolate on Antioxidant Markers In Vivo. Front. Immunol..

[20] Payne M.J., Hurst W.J., Miller K.B., Rank C., Stuart D.A. (2010). Impact of Fermentation, Drying, Roasting, and Dutch Processing on Epicatechin and Catechin Content of Cacao Beans and Cocoa Ingredients. J. Agric. Food Chem..

[21] Peña-Correa R.F., Ataç Mogol B., Fogliano V. (2022). The Impact of Roasting on Cocoa Quality Parameters. Crit. Rev. Food Sci. Nutr..

[22] EUR-Lex-32018R1529-EN-EUR-Lex. https://eur-lex.europa.eu/eli/reg_impl/2018/1529/oj?locale=en.

[23] Pucciarini L., Ianni F., Petesse V., Pellati F., Brighenti V., Volpi C., Gargaro M., Natalini B., Clementi C., Sardella R. (2019). Onion (*Allium Cepa* L.) Skin: A Rich Resource of Biomolecules for the Sustainable Production of Colored Biofunctional Textiles. Molecules.

[24] Puri C., Pucciarini L., Tiecco M., Brighenti V., Volpi C., Gargaro M., Germani R., Pellati F., Sardella R., Clementi C. (2020). Use of a Zwitterionic Surfactant to Improve the Biofunctional Properties of Wool Dyed with an Onion (*Allium Cepa* L.) Skin Extract. Antioxidants.

[25] Rinaldo D., Batista J.M., Rodrigues J., Benfatti A.C., Rodrigues C.M., Dos Santos L.C., Furlan M., Vilegas W. (2010). Determination of Catechin Diastereomers from the Leaves of Byrsonima Species Using Chiral HPLC-PAD-CD. Chirality.

[26] Ianni F., Segoloni E., Blasi F., Di Maria F. (2020). Low-Molecular-Weight Phenols Recovery by Eco-Friendly Extraction from *Quercus* Spp. Wastes: An Analytical and Biomass-Sustainability Evaluation. Processes.

[27] Zafra A., Juárez M.J.B., Blanc R., Navalón A., González J., Vílchez J.L. (2006). Determination of Polyphenolic Compounds in Wastewater Olive Oil by Gas Chromatography-Mass Spectrometry. Talanta.

[28] Proestos C., Komaitis M. (2013). Analysis of Naturally Occurring Phenolic Compounds in Aromatic Plants by RP-HPLC Coupled to Diode Array Detector (DAD) and GC-MS after Silylation. Foods.

[29] Sivilotti P., Falchi R., Vanderweide J., Sabbatini P., Bubola M., Vanzo A., Lisjak K., Peterlunger E., Herrera J.C. (2020). Yield Reduction through Cluster or Selective Berry Thinning Similarly Modulates Anthocyanins and Proanthocyanidins Composition in Refosco Dal Peduncolo Rosso (*Vitis Vinifera* L.) Grapes. Sci. Hortic..

[30] Calderan A., Sivilotti P., Braidotti R., Mihelčič A., Lisjak K., Vanzo A. (2021). Managing Moderate Water Deficit Increased Anthocyanin Concentration and Proanthocyanidin Galloylation in “Refošk” Grapes in Northeast Italy. Agric. Water Manag..

[31] Lisjak K., Lelova Z., Žigon U., Bolta Š.V., Teissedre P.-L., Vanzo A. (2020). Effect of Extraction Time on Content, Composition and Sensory Perception of Proanthocyanidins in Wine-like Medium and during Industrial Fermentation of Cabernet Sauvignon. J. Sci. Food Agric..

[32] Bordiga M., Locatelli M., Travaglia F., Coïsson J.D., Mazza G., Arlorio M. (2015). Evaluation of the Effect of Processing on Cocoa Polyphenols: Antiradical Activity, Anthocyanins and Procyanidins Profiling from Raw Beans to Chocolate. Int. J. Food Sci. Technol..

[33] Wollgast J., Anklam E. (2000). Review on Polyphenols in Theobroma Cacao: Changes in Composition during the Manufacture of Chocolate and Methodology for Identification and Quantification. Food Res. Int..

[34] Suazo Y., Davidov-Pardo G., Arozarena I. (2014). Effect of Fermentation and Roasting on the Phenolic Concentration and Antioxidant Activity of Cocoa from Nicaragua. J. Food Qual..

[35] Zzaman W., Bhat R., Abedin M.Z., Yang T.A. (2013). Comparison between Superheated Steam and Convectional Roasting on Changes in the Phenolic Compound and Antioxidant Activity of Cocoa Beans. Food Sci. Technol. Res..

[36] Żyżelewicz D., Krysiak W., Oracz J., Sosnowska D., Budryn G., Nebesny E. (2016). The Influence of the Roasting Process Conditions on the Polyphenol Content in Cocoa Beans, Nibs and Chocolates. Food Res. Int..

[37] Summa C., Raposo F.C., McCourt J., Scalzo R.L., Wagner K.-H., Elmadfa I., Anklam E. (2006). Effect of Roasting on the Radical Scavenging Activity of Cocoa Beans. Eur. Food Res. Technol..

[38] Tamrin K., Harijono Y., Estiasih S.S., Santoso T.U. (2012). Various Temperature of Vacuum and Conventional Roasting on Color Alteration and Polyphenols Content of Cocoa Powder. J. Food Sci. Eng..

[39] Kendari T. (2012). The Change of Catechin Antioxidant during Vacuum Roasting of Cocoa Powder. J. Nutr. Food Sci..

[40] Ainsworth E.A., Gillespie K.M. (2007). Estimation of Total Phenolic Content and Other Oxidation Substrates in Plant Tissues Using Folin-Ciocalteu Reagent. Nat. Protoc..

[41] Prior R.L., Wu X., Schaich K. (2005). Standardized Methods for the Determination of Antioxidant Capacity and Phenolics in Foods and Dietary Supplements. J. Agric. Food Chem..

[42] Everette J.D., Bryant Q.M., Green A.M., Abbey Y.A., Wangila G.W., Walker R.B. (2010). Thorough Study of Reactivity of Various Compound Classes toward the Folin−Ciocalteu Reagent. J. Agric. Food Chem..

[43] Summa C., McCourt J., Cämmerer B., Fiala A., Probst M., Kun S., Anklam E., Wagner K.-H. (2008). Radical Scavenging Activity, Anti-Bacterial and Mutagenic Effects of Cocoa Bean Maillard Reaction Products with Degree of Roasting. Mol. Nutr. Food Res..

[44] Oracz J., Zyzelewicz D. (2019). In Vitro Antioxidant Activity and FTIR Characterization of High-Molecular Weight Melanoidin Fractions from Different Types of Cocoa Beans. Antioxidants.

[45] Rufián-Henares J.A., Morales F.J. (2007). Functional Properties of Melanoidins: In Vitro Antioxidant, Antimicrobial and Antihypertensive Activities. Food Res. Int..

[46] Zhang H., Zhang H., Troise A.D., Fogliano V. (2019). Melanoidins from Coffee, Cocoa, and Bread Are Able to Scavenge α-Dicarbonyl Compounds under Simulated Physiological Conditions. J. Agric. Food Chem..

[47] Gu L., House S.E., Wu X., Ou B., Prior R.L. (2006). Procyanidin and Catechin Contents and Antioxidant Capacity of Cocoa and Chocolate Products. J. Agric. Food Chem..

[48] Kothe L., Zimmermann B.F., Galensa R. (2013). Temperature Influences Epimerization and Composition of Flavanol Monomers, Dimers and Trimers during Cocoa Bean Roasting. Food Chem..

[49] Stanley T.H., Van Buiten C.B., Baker S.A., Elias R.J., Anantheswaran R.C., Lambert J.D. (2018). Impact of Roasting on the Flavan-3-Ol Composition, Sensory-Related Chemistry, and in Vitro Pancreatic Lipase Inhibitory Activity of Cocoa Beans. Food Chem..

[50] De Taeye C., Kankolongo Cibaka M.-L., Jerkovic V., Collin S. (2014). Degradation of (−)-Epicatechin and Procyanidin B2 in Aqueous and Lipidic Model Systems. First Evidence of “Chemical” Flavan-3-Ol Oligomers in Processed Cocoa. J. Agric. Food Chem..

